# Knowledge, attitudes, and practices on rational antimicrobial use and antimicrobial resistance in Namibia: an online cross-sectional survey

**DOI:** 10.7189/jogh.15.04294

**Published:** 2025-10-24

**Authors:** Anastasia Ndapewa Aluvilu, Walter L Fuller, Aina Ndilimeke Erastus, Frank Busch, Sylvia Dreyer, Lee-Monique Anderson, Juliet Nabyonga-Orem

**Affiliations:** 1 World Health Organization Country Office, UN House, Klein Windhoek, Namibia; 2 World Health Organization Regional Ofﬁce for Africa, Citédu Djoué, Brazzaville, Congo; 3 Institute of International Animal Health/One Health, Friedrich-Loefﬂer-Institut, Greifswald-Insel Riems, Germany; 4 Ministry of Agriculture, Fisheries, Water & Land Reform, Directorate of Veterinary Services, Windhoek, Namibia; 5 Centre for Health Professions Education, Faculty of Health Sciences, North-West University, Potchefstroom, South Africa

## Abstract

**Background:**

Antimicrobial resistance (AMR) is a growing global health threat, particularly in low- and middle-income countries. Understanding public knowledge, attitudes, and practices (KAP) regarding antimicrobial use (AMU) and AMR is essential for designing effective interventions. Through this cross-sectional study, we aimed to assess the KAP related to rational AMU and AMR among Namibian adults using an online, self-administered survey.

**Methods:**

We distributed an online questionnaire between 1 September and 17 November 2024, targeting Namibian adults aged 18 years and older, who had lived in Namibia for at least six months, were willing to participate, and were English literate. We analysed associations between sociodemographic factors and KAP scores using descriptive statistics and χ^2^ tests.

**Results:**

Most of the 541 respondents were female (70%), aged 25–34 (40.5%) and had a tertiary education (91.9%). Good knowledge, attitudes, and practices were observed in 64.3%, 79.7%, and 74.9% of participants, respectively. Higher KAP scores were associated with education, employment, urban residence, and access to water, sanitation, and hygiene infrastructure. Misconceptions persisted across all groups, however, particularly regarding AMU in healthy animals and personal AMR risk.

**Conclusions:**

While surveyed participants demonstrated generally good KAP, gaps remain. Our findings support the need for targeted AMR education campaigns and policy interventions, which need to be supported by a better understanding of the KAP of Namibian adults regarding AMU and AMR.

Antimicrobial resistance (AMR) is a growing global health threat, particularly in low- and middle-income countries, where the burden of infectious diseases is high and access to appropriate treatment is often limited [1]. In 2019, for example, sub-Saharan Africa recorded the highest mortality rate attributable to AMR, at 23.5 deaths per 100 000 population [2]. Key drivers of AMR include the inappropriate use of antimicrobials in human and animal health, poor infection prevention and control, inadequate water, sanitation, and hygiene (WASH) infrastructure, and limited public awareness [1,3,4].

In response to this crisis, the World Health Assembly adopted the Global Action Plan on AMR (GAP-AMR) in 2015, which outlines five strategic objectives, the first of which emphasises improving awareness and understanding of AMR through effective communication, education, and training [5,6]. This has led to initiatives such as the annual World AMR Awareness Week (WAAW), which aims to disseminate accurate, relevant information to the public and media to promote rational antimicrobial use (AMU) [6–8].

Namibia launched its first National Action Plan on AMR (NAP-AMR) in 2019, aligned with the GAP-AMR and guided by six strategic pillars. The fourth pillar focusses on enhancing awareness, collaboration, and communication to reduce misuse and promote rational AMU [9]. Despite this, insufficient knowledge and awareness of AMR and rational AMU among both key stakeholders and the general public – a common phenomenon globally [10,11] – remain significant barriers to progress.

Most studies of knowledge, attitudes, and practices (KAP) on AMU and AMR have focussed on specific groups such as healthcare professionals, farmers, and university students [4,12–14], with few assessing the general public’s understanding [3,10,15], particularly in the African region [11,16] and Namibia [17]. Yet, public knowledge is critical for designing effective interventions, including awareness campaigns, educational initiatives, and policy reforms [3,15,18].

We aimed to fill that gap by assessing the KAP of Namibian adults regarding AMU and AMR. The findings will inform AMR education strategies and support the implementation of Namibia’s NAP-AMR, particularly in addressing persistent misconceptions and tailoring communication strategies to the needs of different population groups.

## METHODS

### Study design, population, and sample size estimation

This was a cross-sectional, online-based study conducted between 1 September and 17 November 2024. It targeted English-literate Namibian residents aged 18 years and older who had lived in the country for at least six months and were willing to participate. Eligibility was assessed through self-reported screening questions at the beginning of the online questionnaire. Participants who did not meet the eligibility criteria were excluded from the study and data analysis, as they were automatically prevented from completing the questionnaire.

We calculated the sample size using OpenEpi, version 3 (Emory University, Atlanta, Georgia, USA), assuming a 95% confidence level and 5% margin of error, resulting in a minimum required sample of 385 participants. 

### Survey instrument and administration

We developed a structured questionnaire based on previously validated KAP tools through a literature review [8,10,11,13–16,19], modifying it to the Namibian context to ensure national relevance and incorporate widely recognised WAAW and AMR/AMU messages [20,21]. 

The questionnaire comprised 55 closed-ended, multiple-choice items, structured across four primary domains: sociodemographic characteristics, knowledge, attitudes, and self-reported practices. An additional complementary section on promotional and educational materials was included, but is not presented here. The KAP sections addressed key topics such as awareness of AMU and AMR, understanding of AMR prevention strategies, antimicrobial use and disposal practices, and hand hygiene behaviours. We first performed a pilot pre-test among 15 participants in August 2024 to assess the clarity and relevance of the survey instrument. We reviewed and used their feedback to refine the questionnaire, but excluded their responses from the final analysis.

The instrument was administered as an anonymous, self-administered online survey using Microsoft Forms (Appendix A of the **Online Supplementary Document**). The link to the final version was disseminated through institutional social media platforms of the Ministry of Health and Social Services, Ministry of Agriculture, Water and Land Reform, the Food and Agriculture Organization, the World Health Organization, the Namibia Institute of Pathology, and the Friedrich-Loeffler-Institut, as well as through personal and professional WhatsApp networks. While we did not systematically track dissemination frequency and reach, the use of these platforms enabled broad circulation across sectors.

### Data cleaning and completeness

All questionnaire items were mandatory, and the online platform was configured to prevent submission of incomplete responses. No manual data cleaning procedures were applied beyond eligibility screening, and no responses were excluded due to data quality concerns.

### KAP scoring methodology

We assessed knowledge items using a three-option format (‘true’, ‘false’, and ‘I do not know’), assigning each correct response one point, and any incorrect ‘I do not know’ responses zero points. Attitude items were evaluated using a five-point Likert scale, with scoring based on alignment with the intended direction of the statement. Responses of ‘strongly agree’ and ‘agree’ for positively worded items were awarded one point, responses of ‘strongly disagree’ and ‘disagree’ for negatively worded items received one point, while neutral responses were scored zero to reflect the absence of a definitive position. Practice items were scored dichotomously: each correct or appropriate response was assigned one point, while incorrect or inappropriate responses received zero.

We analysed scores for KAPs independently to minimise bias and enhance interpretability, calculating aggregated scores across all respondents rather than at the individual level. We standardized each domain score to a scale ranging from 0 to 100 and categorised them using modified Bloom’s cut-off criteria, where scores of ≥80% were classified as good, scores between 60–79% as moderate, and scores <60% as poor [4,12,16,19].

### Data management and analysis

We performed data analysis using Microsoft Excel, version 16.101.3 (Microsoft Corporation, Redmond, Washington, USA) (calculation formulae presented in Appendix B in the **Online Supplementary Document**). Specifically, we summarised data through descriptive statistics and assessed associations between KAP scores and sociodemographic variables using Pearson’s χ^2^ test. All statistical analyses were conducted at a 95% confidence level with a 5% precision level, with *P*-values <0.05 considered statistically significant, *P*-values <0.001 considered highly significant, and *P*-values ≥0.05 considered statistically insignificant [22,23].

## RESULTS

We received 565 responses to the survey, but as 24 participants did not meet the eligibility criteria, only 541 were retained for analysis.

### Sociodemographic characteristics of participants

Of the 541 participating Namibian adults, 381 (70.4%) were female, 219 (40.5%) were aged 25–34 years, 350 (64.7%) were single, 497 (91.9%) had tertiary education, and 378 (69.9%) were employed. Most participants resided in urban areas (n = 448, 82.8%) and were from the Khomas region (46.0%, n = 249). Nearly all participants had access to electricity (n = 512, 94.6%), clean running tap water (n = 522, 96.5%), and a flushing or pit latrine toilet (n = 512, 94.6%). A total of 536 (99.1%) participants reported being exposed to media, with 497 (91.9%) and 383 (70.8%) reporting being exposed to social media and television access, respectively (**Table 1**).

**Table 1:** Sociodemographic characteristics of participants (n = 541)

	n (%)
**Gender**	
Male	158 (29.2)
Female	381 (70.4)
Prefer not to say	2 (0.4)
**Age in years**	
18–24	70 (12.9)
25–34	219 (40.5)
35–44	146 (26.9)
45–54	65 (12.0)
55–64	36 (6.7)
≥65	5 (0.9)
**Marital status**	
Single	350 (64.7)
Married	173 (32.0)
Divorced	10 (1.8)
Widowed/widow	8 (1.5)
**Educational level**	
No formal education	2 (0.4)
Primary	1 (0.2)
Secondary	41 (7.6)
Tertiary	497 (91.9)
**Employment status**	
Employed	378 (69.9)
Self-employed	25 (4.6)
Unemployed	48 (8.9)
Student	77 (14.2)
Pensioner/retired	13 (2.4)
**Region of residence**	
Khomas	249 (46.0)
Erongo	43 (7.9)
Hardap	16 (3.0)
Karas	24 (4.4)
Omaheke	10 (1.8)
Otjozondjupa	10 (1.8)
Kavango West	3 (0.6)
Kavango East	19 (3.5)
Zambezi	11 (2.0)
Oshana	49 (9.1)
Ohangwena	22 (4.1)
Oshikoto	25 (4.6)
Omusati	44 (8.1)
Kunene	16 (3.0)
**Place of residence**	
Urban – within a city/town or in a suburb of a city/town	448 (82.8)
Rural – outside of a city/town, *e.g.* village/farm	93 (17.2)
**Do you live in a household that has electricity**	
Yes	512 (94.6)
No	29 (5.4)
**Do you live in a household that has clean running tap water?**	
Yes	522 (96.5)
No	19 (3.5)
**Do you have access to a flushing/pit latrine toilet?**	
Yes	512 (94.6)
No	29 (5.4)
**Mode of media exposure**	
TV	383 (70.8)
Newspaper	321 (59.3)
Radio	316 (58.4)
Social media (Facebook (Meta), Twitter (X), TikTok, Instagram, *etc*.)	497 (91.9)
No media exposure	5 (0.9)

### KAPs of participants on AMU and AMR

**Figure 1:**
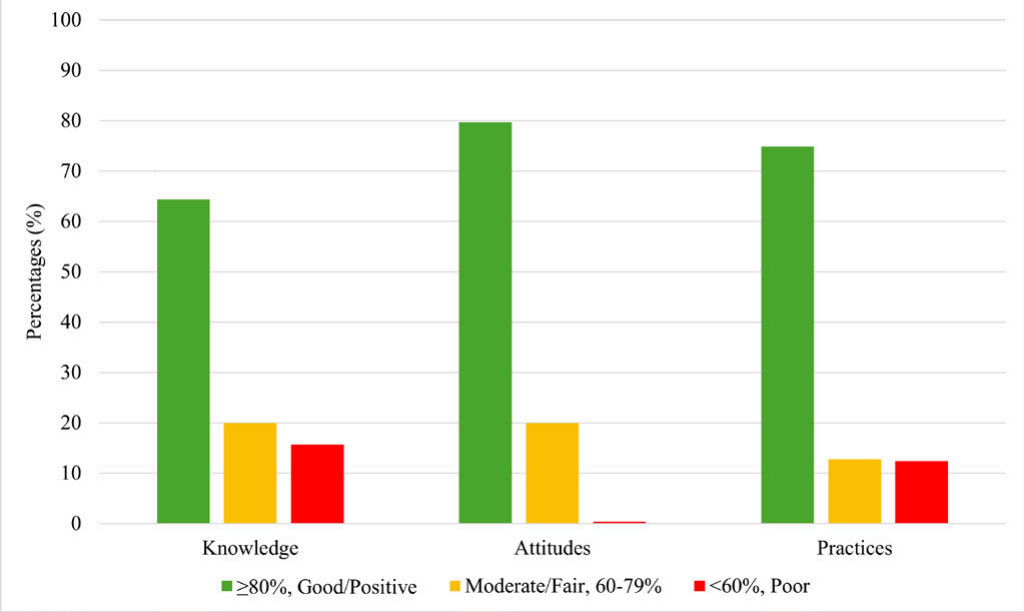
Overall KAP scores towards AMU and AMR (n = 541).

Among the 541 participants, 348 (64.3%) demonstrated good knowledge, 431 (79.7%) showed positive attitudes, and 405 (74.9%) reported good practices (**Figure 1**).

Most participants correctly defined AMR (n = 473, 87.4%) and understood its implications (n = 508, 93.9%). While between 380 (70.2%) and 480 (88.7%) participants correctly identified practices contributing to AMR, fewer of them correctly identified inappropriate AMU scenarios, such as use in healthy animals (n = 285, 52.7%) or use for common illnesses like fever, cough, or sore throat (n = 283, 52.3%) (**Table 2****)**.

**Table 2:** KAP scores for AMU and AMR

Scores and questions (with correct answer in parentheses) for knowledge questions	True	False	I don't know		
Antimicrobial resistance occurs when a microorganism becomes resistant to antimicrobials, and they no longer work as well (true)	473 (87.4)	18 (3.4)	50 (9.2)		
If bacteria are resistant to an antibiotic, it can be very difficult or impossible to treat the infections they cause (true)	508 (93.9)	21 (3.9)	12 (2.2)		
Using antimicrobials when it is not necessary may lead to antimicrobial resistance (true)	457 (84.5)	28 (5.2)	56 (10.4)		
Microorganisms which are resistant to antimicrobials can be spread from person to person (true)	380 (70.2)	72 (13.3)	89 (16.5)		
Antibiotics are effective for any illnesses with fever, cough, cold or sore throat (false)	226 (41.8)	283 (52.3)	32 (5.9)		
Not completing the full course of antimicrobials may cause antimicrobial resistance (true)	480 (88.7)	19 (3.5)	42 (7.8)		
Leftover/unused antimicrobials can be saved for future use or to give someone else (false)	37 (6.8)	473 (87.4)	31 (5.7)		
Antimicrobial resistance is only a problem for people who take antimicrobials regularly (false)	73 (13.5)	400 (73.9)	68 (12.6)		
Resistant microorganisms are only found in hospitals (false)	23 (4.3)	466 (86.1)	52 (9.6)		
Healthy animals can be vaccinated with an antibiotic to prevent future illnesses or help with their growth (false)	188 (34.8)	285 (52.7)	68 (12.6)		
**Scores and questions (with correct answer in parentheses) for attitudes questions**	**Strongly Agree**	**Agree**	**Neutral**	**Disagree**	**Strongly Disagree**
Everyone needs to use antimicrobials responsibly (Strongly Agree)	423 (78.2)	79 (14.6)	29 (5.4)	7 (1.3)	3 (0.6)
I am not at risk of getting an antimicrobial resistant infection, as long as I take my antimicrobials correctly (strongly disagree)	148 (27.4)	132 (24.4)	100 (18.5)	89 (16.5)	72 (13.3)
Parents should make sure all of their children’s vaccinations are up to date (strongly agree)	459 (84.8)	64 (11.8)	12 (2.2)	2 (0.4)	4 (0.7)
People should wash their hands regularly including before handling food (strongly agree)	512 (94.6)	22 (4.1)	4 (0.7)	0 (0)	3 (0.6)
Doctors should only prescribe antimicrobials when they are needed (strongly agree)	456 (84.3)	57 (10.5)	21 (3.9)	4 (0.7)	3 (0.6)
People should only use antimicrobials when prescribed by a doctor (strongly agree)	391 (72.3)	90 (16.6)	45 (8.3)	14 (2.6)	1 (0.2)
Before administering antimicrobials to an animal or animals, you should get consultation from an animal health professional (strongly agree)	414 (76.5)	95 (17.6)	29 (5.4)	3 (0.6)	0 (0)
The use of antibiotics in livestock and crops can increase the presence of resistant bacteria in the environment (strongly agree)	232 (42.9)	111 (20.5)	123 (22.7)	58 (10.7)	17 (3.1)
There is a benefit in using antibiotics in healthy animals (strongly disagree)	68 (12.6)	86 (15.9)	120 (22.2)	123 (22.7)	144 (26.6)
After using antimicrobials in food-producing animals, you should observe the withdrawal period (strongly agree)	332 (61.4)	107 (19.8)	88 (16.3)	11 (2.0)	3 (0.6)
**Scores and questions (with correct answer in parentheses) for practices questions**	**Yes**	**No**			
I take antibiotics to avoid getting a more severe illness, when I suffer from cough and cold (no)	184 (34.0)	357 (66.0)			
I use antimicrobials to speed up the recovery when I get a fever (no)	160 (29.6)	381 (70.4)			
I always complete the course of antimicrobials as prescribed by the doctor (yes)	476 (88.0)	65 (12.0)			
I read the instruction on the label before using medicine (yes)	500 (92.4)	41 (7.6)			
I stop taking antimicrobials when I start feeling better (no)	108 (20.0)	433 (80.0)			
I use the leftover antimicrobials in the event of repeated illness (no)	80 (14.8)	461 (85.2)			
I use antimicrobials only if prescribed by the doctor (yes)	470 (86.9)	71 (13.1)			
If my family member is sick, I would give them my antimicrobials (no)	69 (12.8)	472 (87.2)			
I keep antimicrobials stock at home in case of emergency (no)	112 (20.7)	429 (79.3)			
I use and save my leftover or expired antimicrobials to use on my pets when they get sick (no)	28 (5.2)	513 (94.8)			

A majority of the participants agreed that everyone is responsible for correct AMU (n = 502, 92.7%), parents should ensure children’s vaccinations are up to date (n = 523, 96.7%), and regular handwashing is important (n = 534, 98.7%). Most participants agreed that antimicrobials should be prescribed only when necessary (n = 513, 94.8%) and used only under medical (n = 481, 88.9%) or veterinary guidance (n = 509, 94.1%). However, 382 (70.6%) believed they were not at risk of AMR if antimicrobials were used correctly, and 154 (28.5%) believed antibiotics benefit healthy animals. Additionally, 75 (13.9%) did not believe antibiotic use in livestock and crops contributes to resistant bacteria in the environment.

Lastly, most participants reported completing prescribed antimicrobial courses (n = 476, 88.0%), reading medicine labels (n = 500, 92.4%), not stopping treatment early (n = 433, 80.0%), and not using leftover antimicrobials (n = 461, 85.2%). Additionally, 470 (86.9%) used antimicrobials only when prescribed, 472 (87.2%) did not share antimicrobials with family, and 513 (94.8%) did not keep expired antimicrobials for pets. However, 184 (34.0%) took antibiotics to avoid severe illness from a cough or cold, 160 (29.6%) to speed up recovery from fever, and 112 (20.7%) kept antimicrobials at home for emergencies.

### Association between KAP scores and sociodemographic characteristics

We observed statistically significant associations between KAP scores and several sociodemographic characteristics. Knowledge scores were significantly associated with age (*P* = 0.026), place of residence (*P* = 0.049), and access to WASH infrastructure (*P* = 0.023 and *P* = 0.003) and highly associated with education level (*P* < 0.001) and employment status (*P* < 0.001) (**Table 3**). Participants with tertiary education demonstrated higher knowledge scores (n = 432, 94.7%), and most of the knowledgeable respondents were employed (n = 332, 72.8%). Attitude scores were significantly associated with marital status (*P* = 0.037) and household access to clean water and electricity (*P* = 0.013; *P* = 0.001); and highly associated with age (*P* < 0.001) and employment status (*P* < 0.001). Notably, participants with access to clean running tap water reported more positive attitudes (n = 521, 96.7%). Practice scores were significantly associated with age (*P* = 0.001), region (*P* = 0.024), place of residence (*P* = 0.008) and toilet facilities (*P* = 0.043), while employment status (*P* < 0.001) and household access to electricity (*P* < 0.001) had a very strong link with better practices.

**Table 3:** Association between KAP scores and sociodemographic characteristics

Association between knowledge regarding AMU and AMR and sociodemographic characteristics (n = 541)	≥80%, good (n = 348)	60–79%, moderate (n = 108)	<60%, poor (n = 85)	χ^2^	*P*-value
Gender				11.0334	0.026
*Male*	115 (33.0)	22 (20.4)	21 (25.0)		
*Female*	233 (70.0)	85 (78.7)	63 (75.0)		
*Prefer not to say*	0 (0)	1 (0.93)	0 (0)		
Age				10.637	0.387
*18–24*	37 (10.6)	14 (13.0)	18 (21.4)		
*25–34*	146 (42.0)	44 (40.7)	29 (34.5)		
*35–44*	90 (25.9)	31 (28.7)	25 (29.8)		
*45–54*	45 (12.9)	13 (12.0)	7 (8.3)		
*55–64*	27 (7.8)	5 (4.6)	4 (4.8)		
*≥65*	3 (0.9)	1 (0.9)	1 (1.2)		
Marital status				4.128	0.659
*Single*	220 (63.2)	74 (68.5)	56 (65.9)		
*Married*	113 (32.5)	32 (29.6)	28 (32.9)		
*Divorced*	9 (2.6)	1 (0.9)	0 (0)		
*Widowed/widow*	6 (1.7)	1 (0.9)	1 (1.2)		
Education level				46.517	<0.001
*No formal education*	0 (0)	1 (0.9)	1 (1.2)		
*Primary*	1 (0.3)	0 (0)	0 (0)		
*Secondary*	9 (2.6)	13 (12.0)	19 (22.4)		
*Tertiary*	338 (97.1)	94 (87.0)	65 (76.5)		
Employment status				48.027	<0.001
*Employed*	266 (76.4)	66 (61.1)	46 (54.1)		
*Self-employed*	21(6.0)	2 (1.9)	2 (2.4)		
*Unemployed*	12 (3.4)	18 (16.7)	18 (21.2)		
*Student*	42 (12.1)	19 (17.6)	16 (18.8)		
*Pensioner/retired*	7 (2.0)	3 (2.8)	3 (3.5)		
Region of residence				32.501	0.177
*Khomas*	170 (48.9)	49 (45.4)	30 (35.3)		
*Erongo*	25 (7.2)	11 (10.2)	7 (8.2)		
*Hardap*	10 (2.9)	3 (2.8)	3 (3.5)		
*Karas*	11 (3.2)	7 (6.5)	6 (7.1)		
*Omaheke*	8 (2.3)	1 (0.9)	1 (1.2)		
*Otjozondjupa*	10 (2.9)	0 (0)	0 (0)		
*Kavango West*	3 (0.9)	0 (0)	0 (0)		
*Kavango East*	10 (2.9)	7 (6.5)	2 (2.4)		
*Zambezi*	7 (2.0)	3 (2.8)	1 (1.2)		
*Oshana*	33 (9.5)	5 (4.6)	11 (12.9)		
*Ohangwena*	14 (4.0)	4 (3.7)	4 (4.7)		
*Oshikoto*	16 (4.6)	6 (5.6)	3 (3.5)		
*Omusati*	22 (6.3)	10 (9.3)	12 (14.1)		
*Kunene*	9 (2.6)	2 (1.9)	5 (5.9)		
Place of residence				6.014	0.049
*Urban – within a city/town or in a suburb of a city/town*	298 (85.6)	82 (75.9)	68 (80.0)		
*Rural – outside of a city/town, e.g. village/farm*	50 (14.4)	26 (24.1)	17 (20.0)		
Do you live in a household that has electricity?				4.367	0.113
*Yes*	336 (96.6)	98 (90.7)	78 (91.8)		
*No*	12 (3.4)	10 (9.3)	7 (8.2)		
Do you live in a household that has clean running tap water?				7.578	0.023
*Yes*	343 (98.6)	100 (92.6)	79 (92.9)		
*No*	5 (1.4)	8 (7.4)	6 (7.1)		
Do you have access to a flushing/pit latrine toilet?				11.82	0.003
*Yes*	342 (98.3)	93 (86.1)	77 (90.6)		
*No*	6 (1.7)	15 (13.9)	8 (9.4)		
Media exposure				1.201	0.549
*Yes*	346 (99.4)	108 (100)	84 (98.8)		
*No*	2 (0.6)	0 (0)	1 (1.2)		
**Association between attitudes regarding AMU and AMR and sociodemographic characteristics (n = 541)**	**≥80, positive (n = 431)**	**60–79, moderate (n = 108)**	**<60, negative (n = 2)**	**χ^2^**	***P*-value**
Gender				2.896	0.575
*Male*	132 (30.6)	25 (23.1)	1 (50.0)		
*Female*	298 (69.1)	82 (75.9)	1 (50.0)		
*Prefer not to say*	1 (0.2)	0 (0)	0 (0)		
Age				105.877	<0.001
*18–24*	18 (4.2)	51 (47.2)	1 (50.0)		
*25–34*	183 (42.5)	36 (33.3	0 (0)		
*35–44*	118 (27.4)	27 (25.0)	1 (50.0)		
*45–54*	55 (12.8)	10 (9.3)	0 (0)		
*55–64*	26 (6.0)	10 (9.3)	0 (0)		
*≥65*	4 (0.9)	1 (0.9)	0 (0)		
Marital status				13.387	0.037
*Single*	273 (63.3)	75 (69.4)	2 (100)		
*Married*	146 (33.9)	27 (25.0)	0 (0)		
*Divorced*	9 (2.1)	1 (0.9)	0 (0)		
*Widowed/widow*	3 (0.7)	5 (4.6)	0 (0)		
Education level				8.448	0.207
*No formal education*	2 (0.5)	0 (0)	0 (0)		
*Primary*	1 (0.2)	0 (0)	0 (0)		
*Secondary*	26 (6.0)	15 (13.9)	0 (0)		
*Tertiary*	402 (93.3)	93 (86.1)	2 (100)		
Employment status				38.401	<0.001
*Employed*	320 (74.2)	57 (53.8)	1 (50)		
*Self-employed*	24 (5.6)	1 (0.9)	0 (0)		
*Unemployed*	29 (6.7)	18 (16.7)	0 (0)		
*Student*	52 (12.1)	25 (23.1)	1 (50)		
*Pensioner/retired*	6 (1.4)	7 (6.5)	0 (0)		
Region of residence				29.659	0.282
*Khomas*	203 (47.1)	45 (41.7)	1 (50.0)		
*Erongo*	37 (8.6)	6 (5.6)	0 (0)		
*Hardap*	14 (3.2)	2 (1.9)	0 (0)		
*Karas*	18 (4.2)	6 (5.6)	0 (0)		
*Omaheke*	10 (2.3)	0 (0)	0 (0)		
*Otjozondjupa*	10 (2.3)	0 (0)	0 (0)		
*Kavango West*	3 (0.7)	0 (0)	0 (0)		
*Kavango East*	14 (3.2)	5 (4.6)	0 (0)		
*Zambezi*	9 (2.1)	2 (1.9)	0 (0)		
*Oshana*	36 (8.4)	13 (12.0)	0 (0)		
*Ohangwena*	16 (3.7)	6 (5.6)	0 (0)		
*Oshikoto*	18 (4.2)	7 (6.5)	0 (0)		
*Omusati*	32 (7.4)	12 (11.1)	0 (0)		
*Kunene*	11 (2.6)	4 (3.7)	1 (50.0)		
Place of residence				10.599	0.005
*Urban – within a city/town or in a suburb of a city/town*	368 (85.4)	79 (73.1)	1 (50.0)		
*Rural – outside of a city/town, e.g. village/farm*	63 (14.6)	29 (26.9)	1 (50.0)		
Do you live in a household that has electricity?				8.687	0.013
*Yes*	410 (95.1)	100 (92.6)	2 (100)		
*No*	21 (4.9)	8 (7.4)	0 (0)		
Do you live in a household that has clean running tap water?				13.429	0.001
*Yes*	419 (97.2)	102 (94.4)	1 (50.0)		
*No*	12 (2.8)	6 (5.6)	1 (50.0)		
Do you have access to a flushing/pit latrine toilet?				1.304	0.52
*Yes*	413 (95.8)	97 (89.8)	2 (100)		
*No*	18 (4.2)	11 (10.2)	0 (0)		
Media exposure?				0.345	0.841
*Yes*	429 (99.5)	107 (99.1)	2 (100)		
*No*	2 (0.5)	1 (0.95)	0 (0)		
**Association between practices regarding AMU and AMR and sociodemographic characteristics (n = 541)**	**≥80%, good (n = 405)**	**60–79%, fair (n = 69)**	**<60%, poor (n = 67)**	**χ^2^**	***P*-value**
Gender				4.034	0.401
*Male*	113 (27.9)	19 (27.5)	26 (39.4)		
*Female*	291 (71.9)	50 (72.5)	40 (60.6)		
*Prefer not to say*	1 (0.2)	0 (0)	0 (0)		
Age				30.624	0.001
*18–24*	39 (9.6)	11 (15.9)	20 (29.9)		
*25–34*	167 (41.2)	24 (34.7)	28 (41.8)		
*35–44*	115 (28.4)	19 (27.5)	12 (17.9)		
*45–54*	48 (11.9)	12 (17.4)	5 (7.5)		
*55–64*	33 (8.1)	2 (2.9)	1 (1.5)		
*≥65*	3 (0.7)	1 (1.4)	1 (1.5)		
Marital status				10.05	0.123
*Single*	258 (63.7)	41 (59.4)	51 (77.3)		
*Married*	131 (32.3)	27 (42.0)	15 (22.7)		
*Divorced*	10 (2.5)	0 (0)	0 (0)		
*Widowed/widow*	6 (1.5)	2 (2.9)	0 (0)		
Education level				11.53	0.073
*No formal education*	2 (0.5)	0 (0)	0 (0)		
*Primary*	1 (0.2)	0 (0)	0 (0)		
*Secondary*	22 (5.4)	10 (14.5)	9 (13.4)		
*Tertiary*	380 (93.8)	59 (85.5)	58 (86.6)		
Employment status				46.726	<0.001
*Employed*	302 (74.6)	45 (65.2)	31 (46.3)		
*Self-employed*	18 (4.4)	1 (1.4)	6 (9.0)		
*Unemployed*	32 (7.9)	11 (15.9)	5 (7.5)		
*Student*	42 (10.4)	10 (14.5)	25 (37.3)		
*Pensioner/retired*	11 (2.7)	2 (2.9)	0 (0)		
Region of residence				41	0.031
*Khomas*	201 (49.6)	26 (37.7)	22 (32.8)		
*Erongo*	33 (8.1)	5 (7.2)	5 (7.5)		
*Hardap*	12 (3.0)	1 (1.4)	3 (4.5)		
*Karas*	17 (4.2)	5 (7.2)	2 (3.0)		
*Omaheke*	7 (1.7)	0 (0)	3 (4.5)		
*Otjozondjupa*	8 (2.0)	0 (0)	2 (3.0)		
*Kavango West*	2 (0.5)	1 (1.4)	0 (0)		
*Kavango East*	8 (2.0)	5 (7.2)	6 (9.0)		
*Zambezi*	8 (2.0)	2 (2.9)	1 (1.5)		
*Oshana*	33 (8.1)	7 (1.7)	9 (13.4)		
*Ohangwena*	16 (4.0)	3 (4.3)	3 (4.5)		
*Oshikoto*	18 (4.4)	3 (4.3)	4 (6.0)		
*Omusati*	32 (7.9)	8 (11.6)	4 (6.0)		
*Kunene*	10 (2.5)	3 (4.3)	3 (4.5)		
Place of residence				7.44	0.024
*Urban – within a city/town or in a suburb of a city/town*	345 (85.2)	50 (72.5)	53 (79.1)		
*Rural – outside of a city/town, e.g. village/farm*	60 (14.8)	19 (27.5)	14 (20.9)		
Do you live in a household that has electricity?				9.55	0.008
*Yes*	391 (96.5)	61 (88.4)	60 (89.6)		
*No*	14 (3.5)	8 (11.6)	7 (10.4)		
Do you live in a household that has clean running tap water?				1.355	0.508
*Yes*	395 (97.5)	62 (89.8)	65 (97.0)		
*No*	10 (2.5)	7 (10.1)	2 (3.0)		
Do you have access to a flushing/pit latrine toilet?				6.311	0.043
*Yes*	391 (96.5)	61 (88.4)	60 (89.6)		
*No*	14 (3.5)	8 (11.6)	7 (10.4)		
Media exposure?				2.766	0.251
*Yes*	404 (99.8)	68 (98.6)	66 (98.5)		
*No*	1 (0.2)	1 (1.4)	1 (1.5)		

AMR – antimicrobial resistance, AMU – antimicrobial use

## DISCUSSION

### Knowledge deficits in antimicrobial use and resistance

This study provides important insights into KAP related to AMU and AMR among participating Namibian adults. While most participants demonstrated positive attitudes (n = 431, 79.7%) and responsible practices (n = 399, 74.9%), knowledge levels were comparatively lower (n = 348, 64.3%), indicating an area for improvement. Notably, only 283 (52.3%) of participants correctly identified that antibiotics are ineffective against viral infections, a persistent misconception previously reported in Namibia [17] and across other regions [11,18]. This gap contributes to inappropriate demand for antimicrobials and undermines public health messaging, emphasising the need for targeted educational interventions.

### Prevalent misconceptions and inappropriate antimicrobial use

Despite favourable attitudes, misconceptions remain widespread. A substantial proportion of respondents (n = 380, 70.2%) believed that correct use of antimicrobials eliminates the risk of AMR, reflecting a misunderstanding that may foster complacency and hinder stewardship efforts [18,24]. Additionally, 184 (34.0%) participants reported using antibiotics for colds or coughs to prevent severe illness, indicating persistent irrational use and a lack of understanding of the consequences of unnecessary antimicrobial consumption [17].

### Encouraging attitudes and responsible practices

A key strength observed was the generally positive attitudes and responsible practices among participants. Most respondents (n = 502, 92.8%) acknowledged the collective responsibility in ensuring appropriate AMU and reported avoiding behaviours, such as sharing or storing leftover antibiotics. These findings suggest a comparatively better awareness of AMR’s public health implications and align with those of studies from Bangladesh and Saudi Arabia, which also reported high levels of responsible practices [3,15].

### Limited understanding of agricultural and environmental dimensions

Awareness of AMU in agriculture and its environmental impact was limited. Fewer than half (n = 267, 49.4%) recognised that antibiotic use in healthy animals is ineffective, and only 343 (63.4%) understood the environmental risks associated with such practices. These gaps are concerning given Namibia’s adoption of a One Health approach [25], which emphasises the interconnectedness of human, animal, and environmental health. Similar gaps have been reported in Ethiopia and other sub-Saharan African countries [11,16], underscoring the need to expand AMR education beyond clinical settings to include agricultural stakeholders and the general public.

### Sociodemographic determinants of KAP outcomes

Higher KAP scores were associated with tertiary education, employment, urban residence, and access to WASH infrastructure. These findings are consistent with those of studies from Egypt, South Korea, and other regions [3,10,12,13,15], highlighting the influence of structural determinants on health literacy and behaviour. Older participants also demonstrated more favourable attitudes and practices, likely reflecting accumulated life experience and increased exposure to health information, resulting in more cautious and informed health behaviours [26].

### Leveraging digital platforms for public engagement

Namibia’s digital landscape presents a strategic opportunity to address AMR-related knowledge gaps. With over 1.6 million internet users and a 62.2% internet penetration [27], and 497 (91.9%) of study participants reporting access to social media, digital platforms can be effectively leveraged for education and behaviour change communication. Targeted campaigns *via* social media and television could enhance public engagement and support the goals of the NAP-AMR.

### Study limitations

While this study provides important insights into the KAP related to AMU and AMR among Namibian adults, several methodological limitations should be acknowledged.

One key limitation is that we did not adjust our sample size calculation for anticipated non-response. Future studies should incorporate estimated non-response rates into sample size calculations and use targeted recruitment strategies to improve representativeness. Although we included all regions in the study and disseminated the survey widely to maximise participation, small sample sizes in some regions may limit the generalisability of region-specific findings. We note, however, that we intentionally maintained this granularity to reflect geographic diversity and inform future survey distribution and outreach strategies.

The recruitment strategy, which relied on digital dissemination through institutional social media platforms and WhatsApp networks, likely introduced selection bias, as it may have favoured participation from individuals with internet access and digital literacy, resulting in a sample skewed toward urban, educated, and professionally affiliated respondents. Additionally, the use of an English-only survey may have limited participation from non-English speakers and individuals in rural or underserved areas. To improve inclusivity and rigor, future studies should consider broader dissemination strategies, including mobile-based platforms and translation of survey instruments into local languages.

Another limitation relates to the imbalance in educational attainment within the sample. The overwhelming majority of respondents had tertiary education, with very few participants in other categories, limiting the feasibility of conducting meaningful subgroup comparisons (*e.g.* mean scores or *post-hoc* tests) across educational levels. While the χ^2^ test approach was suitable for testing associations between education and KAP outcomes, future studies with more balanced representation across education levels could explore subgroup differences in greater depth using mean scores and *post-hoc* analyses. Stratified or weighted analysis should also be considered to improve representativeness and analytical rigour. Additionally, incorporating multivariable models and probability-based sampling would enhance both representativeness and analytical robustness.

Furthermore, reliance on self-reported data may not accurately reflect actual behaviours and is subject to recall and social desirability biases. The use of objective measures such as observational methods or structured interviews could help validate self-reported practices and reduce bias. Although we ran a pilot test to assess the clarity and flow of the questionnaire, and while the questionnaire design was informed by previously established and validated tools, we did not perform formal statistical and psychometric validation procedures, which may have affected the reliability of the instrument.

Lastly, these findings represent statistical associations observed within the study sample and should be interpreted in the context of the study’s cross-sectional design, which does not permit causal inference. Nonetheless, the patterns identified may provide useful direction for future research and the design of targeted interventions.

Addressing these limitations will be essential for strengthening future AMR research and informing more targeted interventions.

## CONCLUSIONS

This study provides valuable baseline data on the KAP related to AMU and AMR among Namibian adults. While most participants demonstrated good attitudes and practices, knowledge levels were comparatively lower, underscoring the importance of nuanced public education. The findings also highlight the need for targeted, evidence-based interventions to address persistent gaps in public understanding. Furthermore, the overrepresentation of urban, educated participants suggests that rural populations may be underserved in current AMR awareness efforts.

We recommend several policy actions to address these gaps and strengthen Namibia’s fight against AMR. Public education campaigns should focus on clarifying notable misconceptions regarding AMR risk, the appropriate use of antimicrobials in agriculture, their environmental impact, and community-level factors through a One Health approach. Furthermore, AMR messaging should be extended to rural communities through radio, community health workers, and local leaders to ensure equitable outreach. Integrating AMR education into school and university curricula can also foster early understanding and responsible practices. Strengthening public counselling is likewise necessary to discourage stockpiling and sharing of antimicrobials, with pharmacists playing a key role in consumer education. Digital campaigns should leverage the high media exposure among Namibians, particularly through social media and television, to disseminate engaging and accessible AMR content. Finally, institutionalising regular KAP surveys will enable ongoing monitoring and evaluation, ensuring that interventions remain responsive to emerging trends and population needs. Continued investment in AMR education, surveillance, and stewardship is essential to safeguard public health and promote the rational use of antimicrobials in Namibia.

## Additional material


Online Supplementary Document
