# Supplementary material for: Knowledge, attitudes, and practices on rational antimicrobial use and antimicrobial resistance in Namibia: an online cross-sectional survey
Source: J Glob Health. 2025 Oct 24;15:04294. doi: 10.7189/jogh.15.04294 (PMC12548771; doi:10.7189/jogh.15.04294)
Supplement: Online Supplementary Document [file jogh-15-04294-s001.pdf]

Supplement to: Aluvilu AN, Fuller WL, Erastus AN, Busch F, Dreyer S, Anderson L, Nabyonga-Orem J. Knowledge, attitudes, and practices on rational antimicrobial use and antimicrobial resistance in Namibia: an online cross-sectional survey. J Glob Health. 2025;15:04294.

Online Supplementary Document

1. Appendix A: KAP Questionnaire

Knowledge, Attitudes, and Practices on Antimicrobials Use and Resistance in Namibia

We hope to understand your current understanding, beliefs, and behaviors related to proper use of antimicrobials (AMU) and the development of antimicrobial resistance (AMR), and barriers to seeking education on AMU and AMR, as well as the best way to bring information to you.

*****An antimicrobial is a substance that kills or stops germs like bacteria, viruses, fungi, or parasites from growing. Examples include antibiotics, antifungals, antiparasitics and antivirals.***

The information you provide will be used to guide the designing and developing of interventions and promoting correct use of the antimicrobial agents such as targeted awareness campaigns, educational initiatives, and policy interventions.

Your answers will not be released to anyone and will remain anonymous. Your name will not be written on the survey or be kept in any other records. Your participation is completely voluntary, and you may choose to opt out at any time without facing any consequences.

Lastly, your completion of this survey indicates consent to publish the results of this study.

There are **55 questions**, and it takes about **10 minutes** to complete the survey.

Thank you for your assistance

Section 1

PARTICIPATION CRITERIA

1. Are you 18 years and older? Required to answer. Single choice.
Yes

No

2. Do you currently live in Namibia and have lived in Namibia for at least 6 months at the time of the survey?

Required to answer. Single choice.
Yes

No

3. Are you willing to participate in the survey? Required to answer. Single choice.
Yes

No

Section 2

A. SOCIODEMOGRAPHIC CHARACTERISTICS

4. Gender. Single choice.
Male

Female

Other

Prefer not to say

5. Age. Required to answer. Single choice.
18-24

25-34

35-44

45-54

55-64

65+

6. Marital status. Required to answer. Single choice.
Single

Married

Divorced

Widowed/Widow

7. Education level. Required to answer. Single choice.
No formal education

Primary

Secondary

Tertiary

8. Employment status. Required to answer. Single choice.
Employed

Self-employed

Unemployed

Student

Pensioner/Retired

9. Region of residence. Required to answer. Single choice.
Khomas

Erongo

Hardap

ǁKaras

Omaheke

Otjozondjupa

Kavango West

Kavango East

Zambezi

Oshana

Ohangwena

Oshikoto

Omusati

Kunene

10. Place of residence. Required to answer. Single choice.
Urban - within a city/town or in a suburb of a city/town

Rural - outside of a city/town, e.g., village/farm

11. Do you live in a household that has electricity? Required to answer. Single choice.
Yes

No

12. Do you live in a household that has clean running tap water? Required to answer. Single choice.
Yes

No

13. Do you have access to a flushing/pit latrine toilet? Required to answer. Single choice.
Yes

No

14. Mode of media exposure Required to answer. Multiple choice.
Please select all options that are applicable
TV

Newspaper

Radio

Social media (Facebook (Meta), Twitter (X), TikTok, Instagram etc.)

No media exposure

15. Which animals do you own/handle? Required to answer. Multiple choice.
Please select all options that are applicable
Farm animals (e.g., cattle, pigs, sheep and goats, chicken)

Companion animals (e.g., donkeys and Horses, dogs, and cats)

Aquaculture/Mariculture

Wild animals

None

Section 3

B. KNOWLEDGE QUESTIONS

Do you think the following statements are 'True' or 'False'?

16. Antimicrobial resistance occurs when a microorganism becomes resistant to antimicrobials, and they no longer work as well. Required to answer. Single choice.
True

False

I don't know

17. Antimicrobial resistance occurs when a microorganism becomes resistant to antimicrobials, and they no longer work as well. Required to answer. Single choice.
True

False

I don't know

18. If bacteria are resistant to an antibiotic, it can be very difficult or impossible to treat the infections they cause.

Required to answer. Single choice.
True

False

I don't know

19. Using antimicrobials when it is not necessary may lead to antimicrobial resistance. Required to answer.

Single choice.
True

False

I don't know

20. Microorganisms which are resistant to antimicrobials can be spread from person to person. Required to answer. Single choice.
True

False

I don't know

21. Antibiotics are effective for any illnesses with fever, cough, cold or sore throat. Required to answer. Single choice.
True

False

I don't know

22. Not completing the full course of antimicrobials may cause antimicrobial resistance. Required to answer.

Single choice.
True

False

I don't know

23. Leftover/unused antimicrobials can be saved for future use or to give someone else. Required to answer.

Single choice.
True

False

I don't know

24. Antimicrobial resistance is only a problem for people who take antimicrobials regularly. Required to answer.

Single choice.
True

False

25. Resistant microorganisms are only found in hospitals. Required to answer. Single choice.
True

False

I don't know

26. Healthy animals can be vaccinated with an antibiotic to prevent future illnesses or help with their growth.

Required to answer. Single choice.
True

False

I don't know

Section 4

C. ATTITUDE QUESTIONS

To what degree do you agree with the following statements?

27. Everyone needs to use antimicrobials responsibly. Required to answer. Single choice.
Strongly agree

Agree

Neutral

Disagree

Strongly disagree

28. I am not at risk of getting an antimicrobial resistant infection, as long as I take my antimicrobials correctly.

Required to answer. Single choice.
Strongly agree

Agree

Neutral

Disagree

Strongly disagree

29. Parents should make sure all of their children's vaccinations are up to date. Required to answer. Single choice.
Strongly agree

Agree

Neutral

Disagree

Strongly disagree

30. People should wash their hands regularly including before handling food. Required to answer. Single choice.
Strongly agree

Agree

Neutral

Disagree

Strongly disagree

31. Doctors should only prescribe antimicrobials when they are needed. Required to answer. Single choice.

Strongly agree

Agree

Neutral

Disagree

Strongly disagree

32. People should only use antimicrobials when prescribed by a doctor. Required to answer. Single choice.
Strongly agree

Agree

Neutral

Disagree

Strongly disagree

33. Before administering antimicrobials to an animal or animals, you should get consultation from an animal health professional. Required to answer. Single choice.
Strongly agree

Agree

Neutral

Disagree

Strongly disagree

34. The use of antibiotics in livestock and crops can increase the presence of resistant bacteria in the environment. Required to answer. Single choice.
Strongly agree

Agree

Neutral

Disagree

Strongly disagree

35. There is a benefit in using antibiotics in healthy animals. Required to answer. Single choice.
Strongly agree

Agree

Neutral

Disagree

Strongly disagree

36. After using antimicrobials in food-producing animals, you should observe the withdrawal period. Required to answer. Single choice.
**** withdrawal period is the time you must wait after treating an animal with medicine before you can safely use or eat its products (such as meat, milk, or eggs).*
Strongly agree

Agree

Neutral

Disagree

Strongly disagree

Section 5

D. PRACTICE QUESTIONS

Please answer the following questions on how you use antimicrobials.

37. I take antibiotics to avoid getting a more severe illness, when I suffer from cough and cold. Required to answer.

Single choice.
Yes

No

38. I use antimicrobials to speed up the recovery when I get a fever. Required to answer. Single choice.
Yes

No

39. I always complete the course of antimicrobials as prescribed by the doctor. Required to answer. Single choice.
Yes

No

40. I read the instruction on the label before using medicine. Required to answer. Single choice.
Yes

No

41. I stop taking antimicrobials when I start feeling better. Required to answer. Single choice.
Yes

No

42. I use the leftover antimicrobials in the event of repeated illness. Required to answer. Single choice.
Yes

No

43. I use antimicrobials only if prescribed by the doctor. Required to answer. Single choice.
Yes

No

44. If my family member is sick, I would give them my antimicrobials. Required to answer. Single choice.
Yes

No

45. I keep antimicrobials stock at home in case of emergency. Required to answer. Single choice.
Yes

No

46. I use and save my leftover or expired antimicrobials to use on my pets when they get sick. Required to answer.

Single choice.
Yes

No

Section 6

Section 6

E. PROMOTIONAL AND EDUCATIONAL MATERIALS

The following questions would like to understand the best ways to bring information to you.

47. Have you ever been in any educational campaign regarding antimicrobial use and disposal? Required to answer. Single choice.

Yes

No

48. Have you heard about the World Antimicrobial Resistance Awareness Week (WAAW)?Required to answer. Single choice.

Yes

No

49. Have you seen antimicrobial use (AMU) and antimicrobial resistance (AMR) promotional or educational materials in the following places? Required to answer. Likert.

	Yes	No
Waiting areas of consultation rooms, clinics, outpatient departments, and laboratories		
Hospital wards/units		
Pharmacies		
Retailers of medicinal products		
Other community settings (such as police stations, post offices, schools, etc.)		

50. If you saw them in these places, how will you rate their usefulness in promoting safe antimicrobial use (AMU) and antimicrobial resistance (AMR)?Required to answer. Likert.

Only rate for options you chose **Yes** *in Q.48. If you chose* **No** *for any option in Q.48, please select* **N/A** *here.*

	Not at all useful	Not so useful	Neutral	Very useful	Extremely useful	N/A
Waiting areas of consultation rooms, clinics, outpatient departments, and laboratories						
Hospital wards/units						
Pharmacies						
Retailers of medicinal products						
Other community settings (such as police stations, post offices, schools, etc.)						

51. If you did not see them in those places, how useful do you think they will be in the following places? Required to answer. Likert.

Only rate for options you chose **No** *in Q.48. If you chose* **Yes** *for any option in Q.48, please select* **N/A** *here.*

	Not at all useful	Not so useful	Neutral	Very useful	Extremely useful	N/A
Waiting areas of consultation rooms, clinics, outpatient departments, and laboratories						
Hospital wards/units						
Pharmacies						
Retailers of medicinal products						
Other community settings (such as police stations, post offices, schools, etc.)						

52. Do you own or live in a household with a television (TV)?Required to answer. Single choice.

Yes

No

53. Do you own or live in a household with a radio? Required to answer. Single choice.

Yes

No

54. Do you own a smartphone? Required to answer. Single choice.
Yes

No

55. Do you enjoy listening to jingles, music and melodies? Required to answer. Single choice.
Yes

No

56. Do you enjoy reading daily local newspapers or magazines? Required to answer. Single choice.
Yes

No

57. Do you enjoy reading printed materials, e.g., posters or pamphlets? Required to answer. Single choice.
Yes

No

58. Do you have access to websites or social media, e.g., Facebook? Required to answer. Single choice.
Yes

No

59. How useful do you think these options can be in promoting safe antimicrobial use (AMU) and antimicrobial resistance (AMR) ?

Only rate for questions you chose **Yes** *in Qs.51-57. If you chose* **No** *for any of those questions, select* **N/A** *here.*

	Extremely useful	Very useful	Neutral	Not so useful	Not at all useful	N/A
TV						
Radio						
Smartphone						
Jingles, music and melodies						
Daily local newspapers or magazines						
Printed materials, e.g., posters or pamphlets						
Websites or social media, e.g., Facebook						

2. Appendix B: Microsoft Excel formulae used for the calculations

E_i= (Row Total x Column Total) ÷ Grand Total (N)

Where:

E_i is expected frequency

Row Total is the sum of the observed values in a given row

Column Total is the sum of the observed values in a given column

Grand Total is the sum of all observed values in the entire table.

$$\chi^2 = \sum[(O_i - E_i)^2 \div E_i]$$

Where:

- χ^2 is Chi-squared
- O_i is Observed frequency for the *i*-th category
- E_i is Expected frequency for the *i*-th category

df=(r−1)×(c−1)

Where:

- *r* is the number of rows in the contingency table
- *c* the number of columns in the contingency table

p-value = CHISQ.DIST.RT(chi_squared_statistic, degrees_of_freedom)

3. **Appendix C:** *Research Approval Letter from Ministry of Health and Social Services [MoHSS-Namibia Health Research Ethical Committee (NHREC)]*

REPUBLIC OF NAMIBIA

MINISTRY OF HEALTH AND SOCIAL SERVICES

OFFICE OF THE EXECUTIVE DIRECTOR

Ministerial Building
Harvey Street
Private Bag 13198, Windhoek

Tel: No: 061 -203 2507
Fax No: 061-222 558
Andreas.Shipanga@mhss.gov.na

Ref: 22/4/2/3
Enquiries: Mr. A Shipanga

Date: 15 August 2024

Ms. Anastasia Aluvilu
Un House, 38 Stein Street
Klein Windhoek
Windhoek

Dear Ms. Aluvilu

Title: Knowledge, attitudes and practices on rational use of antimicrobials and antimicrobial resistance among the general population in Namibia: A population-based cross-sectional survey.

1. Reference is made to your application to conduct the above-mentioned study.
2. The proposal has been evaluated and found to have merit.
3. **Kindly be informed that permission to conduct the study has been granted under the following conditions:**
   3.1 The data to be collected must only be used for operational purpose;
   3.2 No other data should be collected other than the data stated in the proposal;
   3.3 Stipulated ethical considerations in the protocol related to the protection of Human Subjects should be observed and adhered to, any violation thereof will lead to termination of the study at any stage;
   3.4 A quarterly report to be submitted to the Ministry's Research Unit;
   3.5 Preliminary findings to be submitted upon completion of the study;
   3.6 Final report to be submitted upon completion of the study;
   3.7 Separate permission should be sought from the Ministry for the publication of the findings.
4. All the cost implications that will result from this study will be the responsibility of the applicant and **not** of the MoHSS.

Yours sincerely,

BEN NANGOMBE
EXECUTIVE DIRECTOR

All official correspondence must be addressed to the Executive Director.

My NAMIBIA
